# Cost-Effectiveness Analysis in Performance Assessments: A Case Study of the Objective Structured Clinical Examination

**DOI:** 10.1080/10872981.2022.2136559

**Published:** 2022-10-17

**Authors:** Zhehan Jiang, Jinying Ouyang, Li Li, Yuting Han, Lingling Xu, Ren Liu, Junhua Sun

**Affiliations:** a Institute of Medical Education, Health Science Center, Peking University, Beijing, Peking, China; b National Center for Health Professions Education Development, Peking University, Beijing, Peking, China; c Department of General Practice, Guangzhou First People’s Hospital, Guangzhou, Guangdong, China; d Psychological Science, University of California Merced, Merced, CA, USA; e Institute of Education, Nanjing University, Nanjing, Jiangsu, China

**Keywords:** Cost-effectiveness, performance assessment, generalizability theory, psychometrics

## Abstract

Medical education assessments are becoming more complex, resulting in the inappropriateness of traditional methods primarily consisting of direct observations, oral examinations, and multiple-choice tests. Advancements in research methods have led to the formation of new modalities, namely performance assessments, which are, on the other hand, always costly in development and implementation. Proposing using the Program Effectiveness and Cost Generalization flow within an assessment context (PRECOG-A), this brief report explores the real financial cost drivers associated with an assessment case in the context of medical education, presents the steps in bridging the effectiveness with its psychometric properties via cost-effectiveness analysis, and evaluates the two-side outcomes for further evaluation decision-making. Referentially providing a framework to investigators and researchers, the illustration of PRECOG-A in this study outlines instructional guidelines for conducting cost-effectiveness analysis in a performance assessment.

## Introduction

A performance assessment (PA) typically involves applying and showing the attribute(s) levels of interest through various performance tasks. PAs are commonly presented with different simulation modalities (e.g., mannequins and task trainers) in a laboratory, clinic, or classroom setting and are credited for evaluating comprehensive skills beyond self- or informant-report. Methodologically, a PA can be regarded as a brief snapshot of a person’s competency in a controlled and unfamiliar environment, and therefore the pitfalls listed in mainstream measurement theories should be considered. This idea is fundamentally reflected in Miller’s Pyramid, of which the higher-level components (i.e., ‘shows how’ and ‘knows how’) are naturally compatible with PAs.

PAs can be regarded as an umbrella term for assessment of performance in both standardized environments and the workplace; The Objective Structured Clinical Examination (OSCE) format, a frequently used PA within a standardized environment in medical education, has test takers rotated through multiple stations where knowledge, skills, and attitudes (KSAs) are assessed. Harden, Lilley, and Patricio described a well-designed OSCE as ‘the gold standard for performance assessment’ [1]. Perhaps a highly known, if not the most, a PA as an OSCE in the field is the Step 2 Clinical Skill of the USA Medical Licensing Examination (USMLE) [2]. An OSCE circumvents existing deficiencies of traditional assessments based on multiple-choice items; it measures how well a test taker can apply KSAs in simulated (and hopefully real) situations, not if they can simply recall the knowledge.

The results of PAs may sometimes be questionable because the tasks assessed contain measurement errors [3]. Measurement errors come from variances such as heterogenous test settings (i.e., raters, items, tasks, and other elements). That said, improper adoptions of simulation modalities and situations prevent the measures from accurately predicting what a person can do in actual workplace settings. No single PA plan fits every evaluation task. However, aligning PAs with measurement theory should yield a clearer picture of the assessment’s quality, indicated presumably by quantified indexes such as reliability.

When it comes to measurement and evaluation, classical test theory (CTT) that decomposes observed scores (i.e., the well-known X = T +E) into true value and measurement errors is almost always a top-notch psychometric framework. The measurement errors, or the E in the CTT’s formula, consist of all unwanted variance/uncertainty but are internally indistinguishable. CTT is unsuitable for PAs as there is always more than one facet. In this scenario, facets are defined as aspects contributing variance to the observed scores, such as raters and tasks/items. Using CTT may oversimplify the scenarios of interest and result in unreliable analyses.

Invented for multi-facet scenarios, on the other hand, generalizability theory (G-theory) can decompose observed scores’ variance into more fine-grained categories and characterize the generalizability (akin to reliability, consistency, or dependability) of PAs [4]. Because of its compatibility with complex designs, G-theory is highly popular in investigating PAs [5,6]. Specifically, it can subsume facets of various (error) variances (e.g., raters, tasks, and test sites) simultaneously, in addition to test takers’ (latent and true) abilities making itself a natural fit for PAs and a ‘conceptual breakthrough’ from CTT [7]. What is more, G-theory contains a D study that allows researchers to generalize the assessment from specific levels of facets to an indefinitely large universe and identify the optimal number of levels of facets to increase generalizability (e.g., using more items/raters in an assessment is more reliable than those that are less). G-theory has been used in the OSCE evaluation to evaluate different sources of variance affecting test takers’ performance, thus giving a detailed reliability diagnosis [8–11].

A detailed introduction to G-theory can be found in Shavelson and Webb [12]. Here, we shortly outline the fundamental components of G-theory and show why it outperforms CTT in the present context. Assume an OSCE consists of two facets- *tasks* and *raters*- that are fully crossed (e.g., each test taker’s performance on each task is rated by all raters). Instead of simply using X = T +E within CTT, one can decompose the performance data to:

$$X_{prt}=\mu +v_{p}+v_{t}+v_{r}+v_{pt}+v_{tr}+v_{pr}+\in_{prt}$$

It indicates that observed performance, $X_{prt}$, for person *p* on task *t* rated by rater *r* is made of the grand mean μ, person effect $v_{p}$, task-facet effect $v_{t}$, rater-facet effect $v_{r}$, interaction effects of any two facets (i.e.,$\;v_{pt}$, $v_{tr}$, and $v_{pr}$), as well as error effect $\in_{prt}$. Different from the E in CTT’s formula, unwanted effects/facets ranging from $v_{t}$ to $\in_{prt}$ (except for the concerned effect $v_{p}$) make up the measurement errors in G-theory framework. After analyzing the G-theory model, the variance of all facets can be estimated, leading towards a series of comparable and interpretable values (i.e., $\sigma_{p}^{2}$, $\sigma_{t}^{2}$, $\sigma_{r}^{2}$, $\sigma_{pt}^{2}$, $\sigma_{tr}^{2}$, $\sigma_{pr}^{2}$, and $\sigma_{prt.e}^{2}$). For example, if the variance of the rater-facet effect $\sigma_{r}^{2}$ is very large, it implies that the rater-consistency is low so that the OSCE provider may consider improving the agreement between raters. The same idea applies to all other facets affecting the quality of the OSCE. What’s more, generalizability/dependability coefficients can be calculated as an overall reliability index in G-theory for the OSCE: $G=\frac{\sigma_{p}^{2}}{\sigma_{p}^{2}+\sigma_{error}^{2}}$ where $\sigma_{error}^{2}$ is defined according to its definition (i.e., relative or absolute). To illustrate, if the $\sigma_{error}^{2}$ is assumed to be relative, we use generalizability coefficient instead of dependability one, reforming the formula to:

G = $\frac{\sigma_{p}^{2}}{\sigma_{p}^{2}+\frac{\sigma_{pt}^{2}}{n_{t}}+\frac{\sigma_{pr}^{2}}{n_{r}}+\frac{\sigma_{ptr}^{2}}{n_{r}*n_{t}}}.$

The n s and their scripts indicate the total level number of the corresponding facet. It is evident that manipulating n s can affect the value of *G* estimate, which is exactly what a D study attempts to achieve: what would the coefficient be if one or multiple facets’ levels are changed without actually altering them? That said, given a set of one n s, one can estimate a new *G* for the set (i.e., a plan) and, therefore, compare the changes if there are different plans.

Like all other PAs, OSCEs consume tremendous resources and time for their development and implementation [13]. Ideally, to make an OSCE a good form of assessments, five criteria described by Van der Vleuten (i.e., reliability, validity, cost-efficiency, educational impact and acceptability) should be met [14]. In practice, although excelling in all criteria would be perfection, pragmatically there often has to be compromised [15]. As the title indicates, this report addresses the first three aspects of the criteria: it associates PAs’ costs and quality, which is reflected as reliability and validity, while reliability is also referred to as internal validity or internal structure of the assessment tool [16]. Presumably, OSCE providers should spare no effort to improve PA quality, but budget limits are almost always determining the ceiling of quality improvement. Finding a balance between financial capacity and assessment setups is critical to the assessment providers. A quantitative investigation of this ‘trade-off’ is termed a cost-effectiveness analysis (CEA), widely known in health economics [17]. This brief report compares a cost reduction proposal with its current plan. A CEA for the OSCE is presented to show the flows of bridging the costs with its psychometric properties; it provides a reference for researchers to decide on PA investment.

## Methods

**Figure 1.**
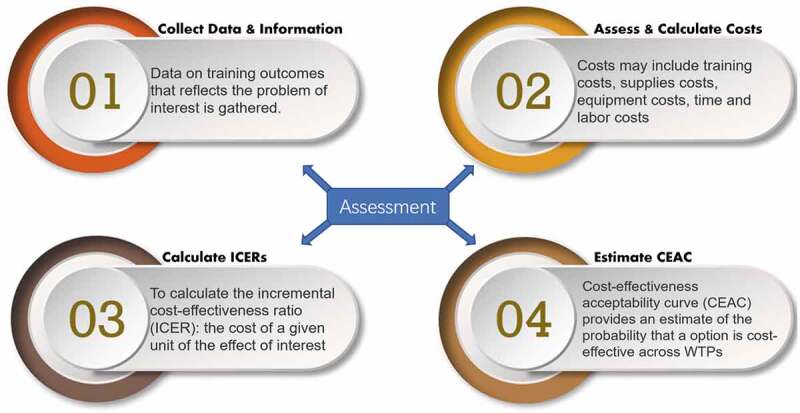
The four steps of program effectiveness and cost generalization within an assessment context.

This brief report adopts an OSCE case from Jiang and his colleagues [10], where performance data and corresponding costs were recorded. As seen in Figure 1.

The Program Effectiveness and Cost Generalization (PRECOG [18];) flow that consists of four steps was used to conduct the CEA. The PRECOG was initially proposed for CEAs in health profession teaching and training, which substantially involve the OSCE during the educational cycles. The PRECOG was initially proposed for CEAs in health profession teaching and training, which substantially involve the OSCE during the educational cycles. The PRECOG underlines the need for models that should guide educators, administrators and decision-makers in prioritizing educating programs; its four steps represent ‘increasing levels of recommendation strength, from assessment of cost-effectiveness and cost to generalization of cost-effectiveness evidence’. The 2nd step, particularly, sets applications to OSCE scenarios where the cost categories are highly OSCE-specific. Since its introduction, the PRECOG has been adopted in many OSCE-related works [19–21].

Because effectiveness is always not monetary in a CEA, one should define the maximum amount decision-makers are willing to pay to achieve or avoid a certain outcome; this is called willingness to pay (WTP). A simple illustration of WTP can be a question asking, ‘what would be the maximum you would have been willing to pay for this course?’ [22]. A proposed plan or intervention should only be adopted if the cost of effectiveness is less than WTP. This report differs from other PRECOG studies because the context – assessment demands theoretical and methodological support from the educational measurement field, meaning that the effectiveness analysis should be placed within an assessment context (PRECOG-A). A key concept in a CEA is the incremental cost-effectiveness ratio (ICER), which evaluates the difference in costs and outcomes between a reference intervention (known as the base case) and the alternative(s). Mathematically, the ICER is equal to (cost of intervention – cost of base case)/(outcome of intervention – outcome of base case).

Despite the G-theory analysis being based on real performance, there were also data points simulated via the CEA, which deployed the Monte Carlo mechanism to support decision-making when uncertainty was assumed to contribute in specific ways. The core of the Monte Carlo mechanism is using computational sampling to ‘mimic’ possible values under some complex scenarios that can be represented as complex statistical distributions.

The OSCE contained 4 exam forms; each form was delivered at 18 test stations (i.e., 10 history-taking, 3 physical exam, 1 diagnosis and clinical management, 1 radiographic interpretation, 1 laboratory studies interpretation, and 1 critical appraisal of research works) from several mandatory specialty tracks (i.e., KSA domains such as pediatrics and gynecology). To tally up, 72 unique form-based stations (4 forms * 18 stations) were established in a test site, while there were six sites, one of which test takers were randomly assigned to. The setting in G-theory could be outlined as *person: site x form x station*, meaning the test takers were nested within the sites, where the sites, the forms, and the stations are fully crossed.

As the OSCE was a summative end-of-career graduating exam for selecting qualified candidates against certain entry standards (i.e., everyone about a determined bar/threshold receives a pass), the absolute-error-based generalizability coefficient was calculated [23].

## Results

The detailed calculation and analysis are documented in the Appendix. After using a G-theory model, the $E\rho_{\Delta }^{2}$ reached at 0.8. At this stage, all the information essentially completed the first step of the PRECOG-A.

The second step cumulated and classified the costs according to the OSCE’s design. Costs for OSCEs consist of multiple subjects, including station development, question writing and reviews, rater training, administration, technician support, scoring, exam board meeting, and many others. As the previous step deployed G-theory models conditioning on facets such as sites, test forms, and stations, the costs were aggregated correspondingly such that the unit amounts were £15,896 per site, £6,677 per version, and £4,843 per station, resulting in the total cost of £209,240.

Three facets (i.e., *sites, forms*, and *stations per track*) were proposed to change to 5, 2, 17 by the OSCE providers; that said, we ran a D study reducing each of site, form and station by one, leading to a total cost of £147,491 and an $E\rho_{\Delta }^{2}$ of 0.75. The ICER, £1,029,150, was obtained by the ratio of the difference in costs between facet combinations to the difference in effectiveness, that is, (£209,240-£147,491)/(0.81–0.75); To make the interpretation more reasonable, the denominator scale could be converted to ‘0.01 change in $E\rho_{\Delta }^{2}$ (up to 1)’ and therefore, the ICER was £10,292.

**Figure 2.**
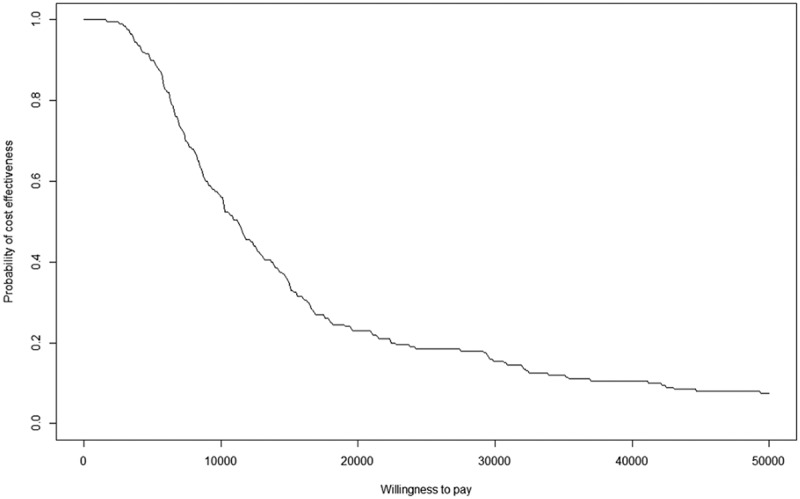
Cost-effectiveness acceptability curve.

The Monte Carlo simulations were used to incorporate uncertainty into the cost-effectiveness acceptable curve (CEAC) construction. The variances of the $E\rho_{\Delta }^{2}$ and the costs were obtained from mathematical formulas and the 10-year consumer price index, respectively [24]. The WTPs were converted to the same scale as the aforementioned ICER and the trends are shown in Figure 2.

For WTP values above £5,000 per $0.01\times E\rho_{\Delta }^{2}$, there was a 95% probability that proposal was the more cost-effective than the current plan.

## Discussion and Conclusion

Based on Consensus Statement on Performance Assessment in 2011 [25], the board of Ottawa Conference 2020 published best practice recommendations for OSCEs, emphasizing evidence-based logic such as interpreting and utilizing test scores more holistically in the decision-making process [26]. The scoring-generalization-extrapolation-implications cycle was proposed to justify decisions [27], leading toward a programmatic assessment model aiming at optimizing the decision function of assessment. It is self-evident that any ‘optimization’ is meaningless without considering resource constraints and/or limits. This report focuses on optimizing the settings of PAs, of which systematic evaluation has received increasing attention during the past years [26,28]. We provide a methodological demonstration for conducting CEAs to meet the calls for economic evaluations in PAs. The 4-step PRECOG-A offers a clear quantitative reference for further decision-making from the perspective of the cost-effectiveness trade-off. Although our demonstration is rooted in a local environment, the reference can be generalized to different scales, such as large-scale licensing exams organized by corresponding national associations or boards. What D-study plays in the PRECOG-A was that it provides a quantitative prediction to the change of generalizability, a primary PAs’ quality index, when the setting is altered

Walsh and colleagues claim that ‘medical education is expensive’ [29], and various strands of studies have investigated the effectiveness, utility, and acceptability of different medical education interventions and programs, resulting in more CEA in medical education [30,31]. However, PAs within an appropriate psychometric framework is rarely set as the central topic in the CEA literature, yet they play a critical role in medical education. Therefore, this report makes a difference by aligning two aspects – PAs’ psychometric properties and CEA – together to broaden the research arena of this kind. G-theory is integrated into CEA to estimate the expenditure of facets’ cost when varying the unit of generalizability ($0.01\times E\rho_{\Delta }^{2}$) and the ICER is needed, where the balance in between always ends up into a decision-making problem. Given a specific budget constraint, researchers can optimize the PA configuration to meet a sufficient reliability level; This demonstrates appealing features that applying G-theory only can’t offer. In this report, increasing WTP makes the current proposal less attractive. The final decision, of course, should be based on selecting an appropriate WTP, which is somehow abstract and difficult.

Interpreting CEA results of PRECOG is always challenging, no matter which context is referred to. In the present scenario, the first step is more than simple data collection: investigators should learn the design thoroughly so that later steps’ modeling strategies can be appropriately chosen. The second step heavily consumes time and effort in organizing expenditures and defining unit costs for categories/facets that can be altered. It is highly recommended to involve quantitative experts in the third step: the modeling for both the performance data and the costs matter significantly to the analysis. For instance, instead of using G-theory models, one can choose item response models for rating scales to handle the same inquiry, although the choice may not be optimal. What makes Steps 3&4 the hardest among the PRECOG-A is defining a threshold between monetary and psychometric units: both WTP and CEAC rely on the threshold, which in the present context does not have standard references. The decision-makers may deploy the Delphi method and other similar consulting efforts to draw appropriate values for the final decision.

Limitations also exist, for example, $E\rho_{\Delta }^{2}$ was the only effectiveness and modeling processes were simplified to a certain degree. In practice, researchers are required to be more comprehensive in evaluating PAs against validity standards, such as internal structure and response process for OSCEs [27,32,33]. That said, a decision about whether a PA possesses the validity evidence to support inferences is supposed to be holistic; Adjusting a number on increments of generalizability is merely a slice. As seen in tremendous published results, it is highly challenging to boost an OSCE above 0.85 unless the testing time is substantially longer, possibly causing overwhelming cognitive burdens for test takers [34]. Realistically, the decision should always be concerned with a broader picture, including blueprinting, threats to scoring in Kane’s model, and other psychometrics properties.

It is also a significant limitation that the performance data and the costs do not originate from the same research agenda. It is uncertain if step 2 of PRECOG was correctly performed in order to proceed with the computation of ICER. When it comes to a high-stake decision-making practice, it would be crucial to demonstrate that main expenses have been considered and the cost analysis has been completed properly.

Future applied studies should comprehensively collect evidence with an integration of relevant elements from both theories and practices, grounded an umbrella perspective.

## Appendix: Detailed Calculation and Analysis

##Known parameters from

var_pvs = var_p = 17.652

var_s = 0

var_v = 0.737

var_sv = 0.867

var_e = 42.157

var_ev = 38.968

var_es = 46.631

var_evs = 34.692

var_pevs = 187.374

var_p+ var_s+ var_sv+var_e+ var_ev+var_es+var_evs+var_pevs+var_pvs

g_coef<-function(n_v,n_s,n_e){

var_pvs = var_p = 17.652

var_s = 0

var_v = 0.737

var_sv = 0.867

var_e = 42.157

var_ev = 38.968

var_es = 46.631

var_evs = 34.692

var_pevs = 187.374

var_p/(var_p+var_pvs/(n_v*n_s)+var_pevs/(n_e*n_v*n_s))

}

phi_coef<-function(n_v,n_s,n_e){

var_pvs = var_p = 17.652

var_s = 0

var_v = 0.737

var_sv = 0.867

var_e = 42.157

var_ev = 38.968

var_es = 46.631

var_evs = 34.692

var_pevs = 187.374

var_p/(var_p+var_s/(n_s)+var_v/(n_v)+var_sv/(n_s*n_v)+var_e/(n_e)+var_ev/(n_e*n_v)+var_es/(n_e*n_s)+var_evs/(n_e*n_s*n_v)+var_pevs/(n_e*n_s*n_v))

}

g_coef(4,6,18)

phi_coef(4,6,18)

## Specify costs for each facet

cost_s<-15,896

cost_v<-6677

cost_e<-round((6577 + 3108)/2,0)

## Use this function to optimize the model. The three parameters are

## evaluated on given their range and scope.

totalcost_unlist_phi <- function(x) {

n_v<-round(x[1]);n_s<-round(x[2]);n_e<-round(x[3])

if(phi_coef(n_v,n_s,n_e)>Threshold){penalty<-1}else{penalty<-50}

(cost_s*n_s+ cost_v*n_v+ cost_e*n_e)*penalty

}

#Original Cost

totalcost_unlist_phi(c(4,6,18))

SD_g<-function(x,n){

sqrt(x*(1-x)/n)

}

ou1<-rnorm(200,0.81,SD_g(0.81,278))

ou2<-rnorm(200,0.75,SD_g(0.81,278))

ou<-cbind(ou2,ou1);ou<-ou*100

#10 Year’s CPI

CPI<-rbind(

c(2020,108.2, 108.6, 108.6, 108.5, 108.5, 108.6, 109.1, 108.6, 109.1, 109.1,108.9, 109.2, 108.8),

c(2019, 106.3, 106.8 ,107.0, 107.6, 107.9, 107.9, 107.9, 108.4, 108.5, 108.3, 108.5, 108.5, 107.8),

c(2018, 104.4 ,104.9 ,105.0, 105.4, 105.8, 105.8, 105.8, 106.5, 106.6, 106.7, 107.0 ,107.1, 105.9),

c(2017, 101.4 ,102.1 ,102.5, 102.9, 103.3, 103.3, 103.2, 103.8, 104.1, 104.2, 104.6 ,104.9, 103.4),

c(2016, 99.6, 99.8, 100.2, 100.2, 100.4, 100.6, 100.6, 100.9, 101.2, 101.2, 101.5, 101.9, 100.7),

c(2015, 99.3, 99.5, 99.7, 99.9, 100.1, 100.2 ,100.0, 100.3, 100.2, 100.3, 100.3, 100.3, 100.0),

c(2014, 99.0, 99.5, 99.7, 100.1, 100.0, 100.2,99.9, 100.2, 100.3, 100.4, 100.1, 100.1, 100.0),

c(2013, 97.1, 97.8, 98.1, 98.3, 98.5, 98.3, 98.3, 98.7, 99.1, 99.1, 99.2, 99.6, 98.5),

c(2012, 94.6, 95.1, 95.4, 96.0, 95.9, 95.5, 95.6, 96.1, 96.5, 97.0, 97.2, 97.6, 96.0),

c(2011, 91.3, 92.0, 92.2, 93.2, 93.4, 93.3, 93.3, 93.8, 94.4, 94.5, 94.6, 95.1, 93.4),

c(2010, 87.8, 88.2, 88.7, 89.2, 89.4, 89.5, 89.3, 89.8, 89.8, 90.0, 90.3, 91.2, 89.4)

co1<-rnorm(200,209,240,209,240*sd(CPI[,-1])/100)

co2<-rnorm(200,147,491,147,491*sd(CPI[,-1])/100)

co<-cbind(co2,co1)

he <- BCEA::bcea(ou, co)

ceac.plot(he)
